# Evaluation of Impact Strength and Flexural Strength of Polyether Ether Ketone vs. Computer-Aided Design/Computer-Aided Manufacturing Polymethyl Methacrylate Denture Base Materials: An In-Vitro Study

**DOI:** 10.7759/cureus.47929

**Published:** 2023-10-29

**Authors:** Tushar, Priya Rani, Ananya, Surender Kumar, Jayant Prakash, Jayaprakash M B.

**Affiliations:** 1 Prosthodontics, Dental College, Rajendra Institute of Medical Sciences, Ranchi, IND; 2 Dentistry, Sadar Hospital, Muzaffarpur, IND

**Keywords:** denture base resin, flexural strength, impact strength, cad/cam, pmma, peek

## Abstract

Objective: The objective is to comparatively assess the impact strength and flexural strength of polyether ether ketone (PEEK) vs. computer-aided design/computer-aided manufacturing (CAD/CAM) polymethyl methacrylate denture base material.

Methods: A total of 90 samples were fabricated with traditional heat cure PMMA, PEEK, and CAD/CAM PMMA and divided into three groups of 30 samples each. The impact strength of all the samples was measured using an Izod impact tester with a pendulum in the air at 23±2°C. A three-point bending test was used in a Universal Testing Machine to assess the flexural strength of all the samples. The impact strength and flexural strength mean values were computed using a one-way ANOVA test.

Result: Impact strength and flexural strength of PEEK (IS=10.22±1.25 kJ/m^2^ and FS=120±8.0 MPa) is almost identical to CAD/CAM PMMA sample (IS=9.595±3.313 kJ/m^2^ and FS=118.11±5.00 MPa) whereas for conventional heat cure PMMA (IS=4.00±.011 kJ/m^2^ and FS=75.4±4.50 MPa) the values are least among the three.

Conclusion: PEEK or CAD/CAM PMMA share almost identical and superior mechanical properties, and both can be used as better alternatives for complete denture fabrication rather than using conventional heat cure PMMA.

## Introduction

Complete denture prostheses are the most traditional and time-honored form of prosthodontic treatment preferred by edentulous patients for ages. Various denture base resin materials have been invented and are being used in dentistry to fabricate complete dentures. Polymethyl methacrylate (PMMA) is the most accepted and universally utilized denture base material for the fabrication of complete dentures, introduced in the year 1937 by Dr. Walter Wright [1]. Despite being one of the famous materials with excellent physical and mechanical properties, it has certain drawbacks because of which there is a requirement for enhancement in the strength of PMMA. Impact and flexural forces are the major cause of complete denture fracture [2]. Uneven forces acting on the denture base due to irregular alveolar ridge led to a focus on flexural strength. Microscopic cracks generally develop at a stress concentration area due to flexural fatigue. These types of cracks do not develop in a single application of forces but occur throughout continuous forces [3]. Good flexural strength of denture base resins determines excellent fracture resistance, which ensures less denture failure. Impact strength is determined by the energy that solid substances absorb when sudden blows strike them; hence denture base resins always need to have excellent impact strength in order to prevent breakage [4]. Therefore, flexural strength, as well as impact strength of denture base resin materials, has been utilized to compare their performance. Methods of fabrication play a crucial role in determining the impact strength of dentures. Conventional methods of fabrication produce various internal defects such as porosity, which leads to a certain amount of shrinkage which directly affects the mechanical properties of dentures [1]. To overcome the problems associated with conventional fabrication techniques, the computer-aided design/computer-aided manufacturing (CAD/CAM) technique was introduced which produces dentures by milling pre-polymerized discs of resin material [5]. These discs are produced under high heat and pressure thus reducing the chances of shrinkage and porosity due to residual monomer content [6]. CAD/CAM technique uses both PMMA resins and polyether ether ketone (PEEK) material for fabrication [6,7].

PEEK (-C6H4-OC6H4-O-C6H4-CO-)n is a semi-crystalline linear polycyclic aromatic polymer. Few English scientists developed PEEK in 1978 which was later commercialized [8,9]. PEEK was initially introduced in the field of aerospace engineering and then used for spinal and hip implants in 1999 because of its excellent biological and chemical inertness. It was observed and well documented in the literature that PEEK absorbed less water and posed no or less shrinkage compared to PMMA [9]. However, there are fewer details in the literature regarding impact strength as well as flexural strength of CAM/CAD PMMA vs. PEEK. Thus, this study aims to determine the impact strength as well as flexural strength of CAM/CAD PMMA, PEEK, and traditional heat cure PMMA.

## Materials and methods

Sample fabrication

Samples were divided into three groups as per the material used in fabrication for complete denture - Group I (n=30) - Conventional heat cure PMMA, i.e., the control group, Group II (n=30) - CAD-CAM PMMA, and Group III (n=30) - PEEK.

Conventional Heat cure PMMA samples were fabricated utilizing a metallic mould of size 65mm×10mm×3mm, In the mould, heat cure powder polymer and liquid monomer were mixed as per manufacturer’s instruction using compression molding technique (4.4mL monomer, 10g powder) (Lucitone, Dentsply Sirona US).

**Figure 1:**
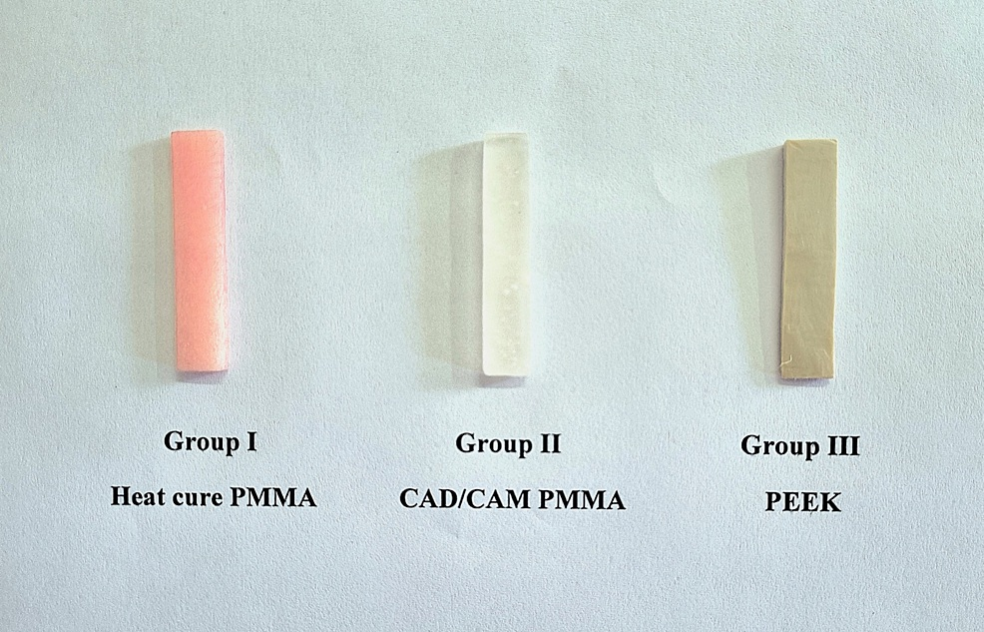
Finished and polished Heat cure PMMA, CAD/CAM PMMA & PEEK Samples

CAD-CAM PMMA discs (Dentsply Sirona US, multilayer PMMA Disc) of 98mm×25mm & PEEK discs (InterDent CC DISK PEEK) of 1414mm×20mm were scanned and milled using a 5-axis milling machine (Imes-icore -350i Gmbh, Germany) with size of 65mm×10mm×3mm. A total of 60 samples were obtained from the milling machine and were subjected to finishing and polishing using silicon carbide paper with different grits along with pumice powder. All the samples prepared (Figure 1) were stored in artificial saliva at 37 degrees centigrade for 30 days and then subjected to testing procedures.

Testing of samples

**Figure 2:**
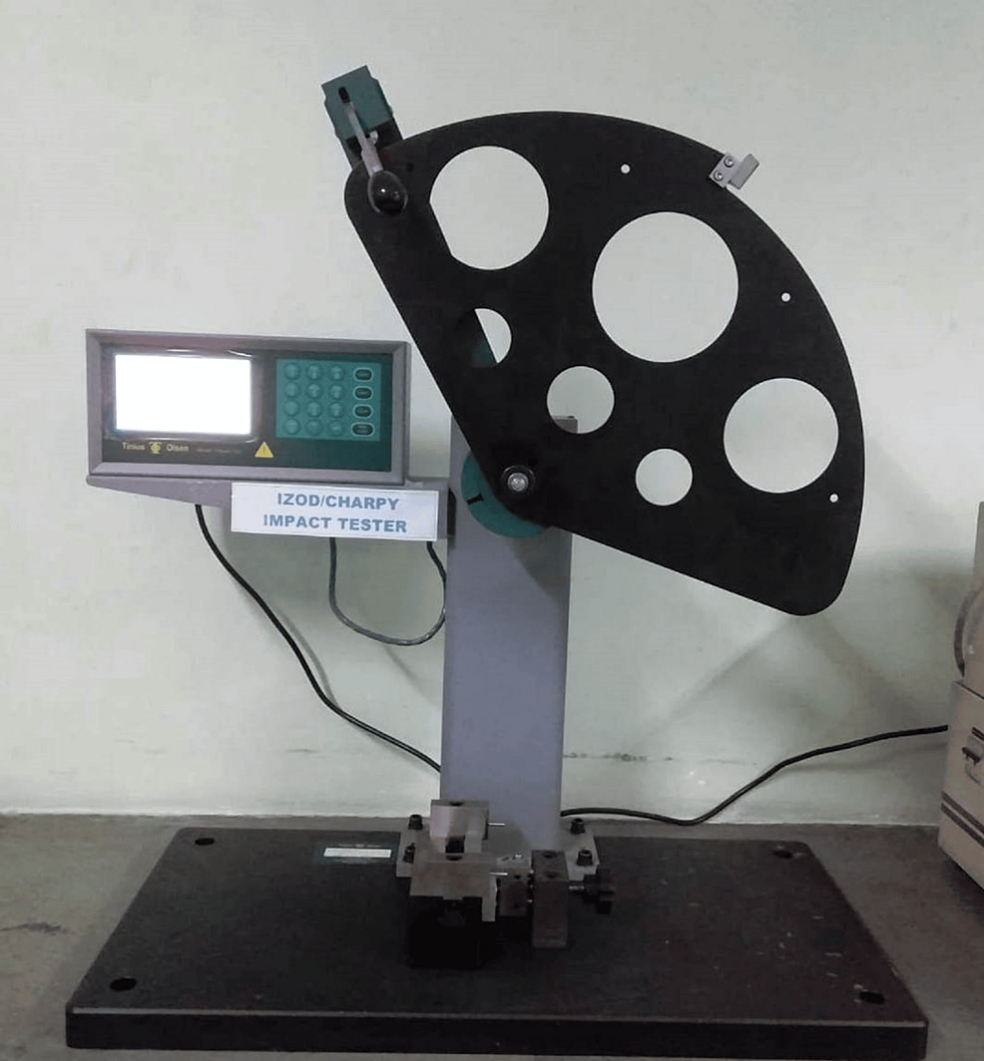
Izod Impact Tester

Impact Strength values of all the samples were measured using an Izod impact tester (Figure 2) having a pendulum suspended in the air at 23±2ºC. Zeroing of the pendulum was done before testing of sample, i.e., Air resistance was recorded when free swing was done, and the pointer was stabilized.

The impact strength was computed with the following formula:

Impact strength = U/a(b-e)

Where a stands for the width of the sample, b is the thickness and e is the depth of the notch.

**Figure 3:**
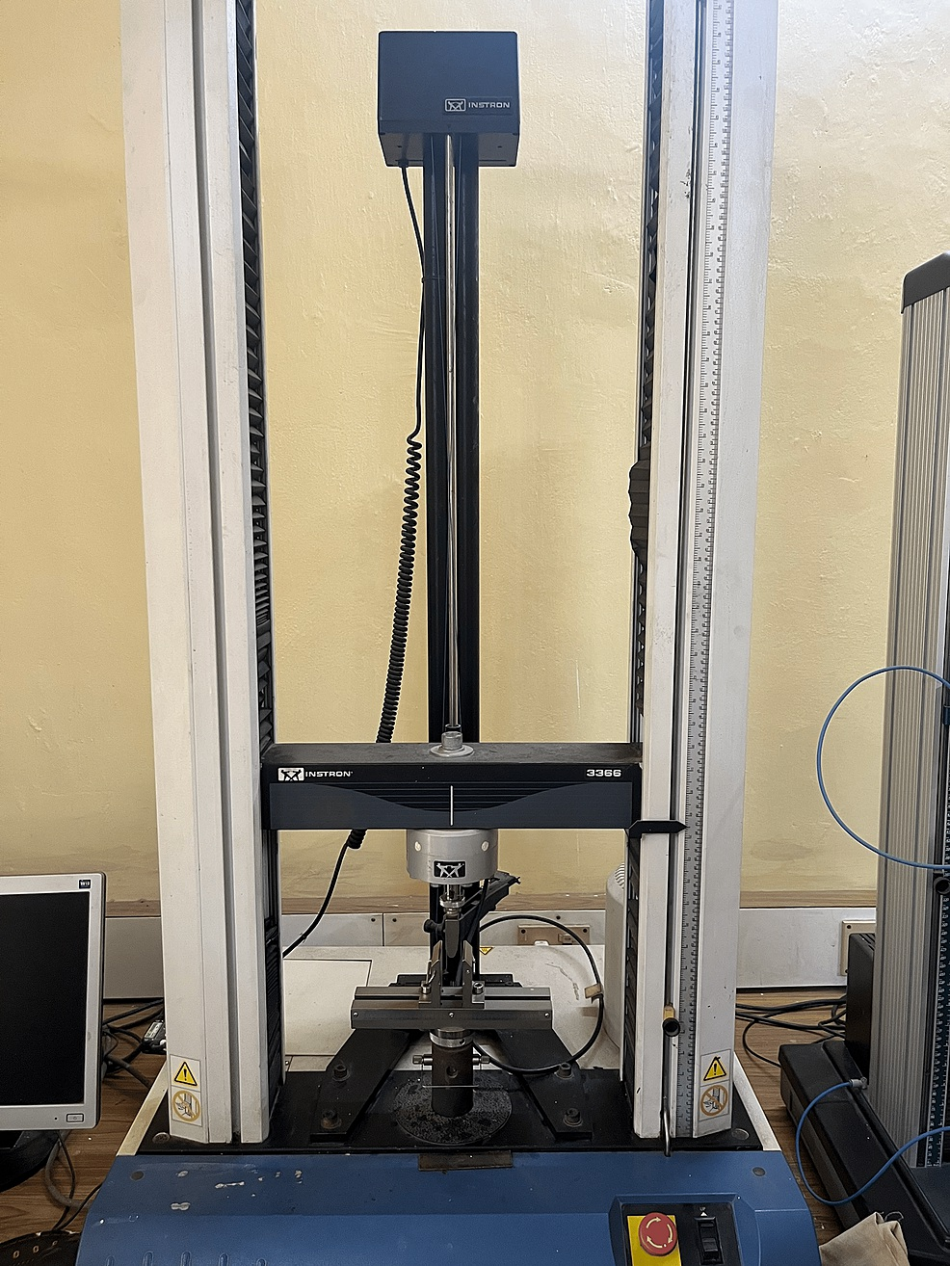
Universal Testing Machine

Flexural strength values of all the samples were measured utilizing a three-point bending test at the universal testing machine (Figure 3) (Instron®, US). It is a machine to test the strength of bars supported at both ends, subject to loading. The device contains a loading wedge and an adjusting wedge placed 50 mm apart. Samples were placed at the center of wedges and loading wedges travelled at a speed of 5mm/minute with 500kgf load. Data were calculated by the program of the UTM machine.

The flexural strength (σ) was computed with the following formula: 

S = 3FI/2bd2

Where F stands for the maximum force applied, I is the distance between two wedges, b is the breadth of the sample and d is the depth of the sample.

Statistical analysis

The collected data were combined in an MS Office Excel sheet (version 2013, Redmond, Washington, USA) and statistical analysis was performed utilizing the Statistical Package for Social Sciences (SPSS version 21.0, IBM Corp., Armonk, NY, USA). The one-way ANOVA test was utilized to assess the samples' average values for the impact strength as well as flexural strength. Tukey pairwise comparisons were used for the intergroup comparison (Tukey simultaneous tests for differences of means).

## Results

**Figure 4:**
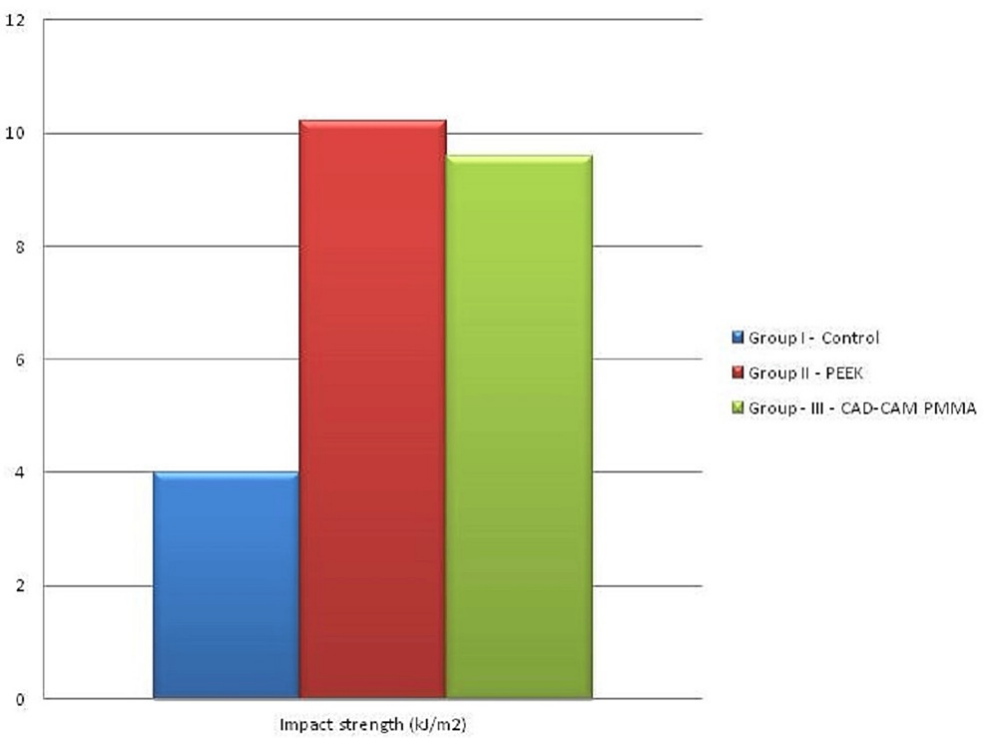
Mean impact strength values (kJ/m2)

**Figure 5:**
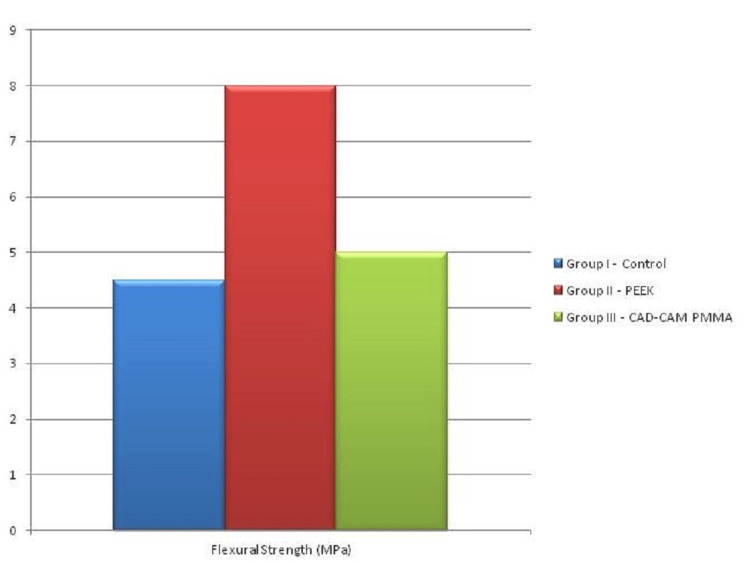
Mean flexural strength values (MPa)

Ninety samples were fabricated and assessed for impact strength and flexural strength (30 samples for a test per group). The mean value of the impact strength of samples was assessed using one-way ANOVA. The mean value of Impact strength of conventional Heat cure PMMA was 4.00±.011 kJ/m^2^ which was the least among the three-sample group and the mean value of CAD/CAM PMMA was 9.5±3.313 kJ/m^2^ which was almost identical to the mean value of PEEK 10.22±1.25 kJ/m^2^ (Table 1, Figure 4). The mean value of flexural strength for all samples was assessed by one-way ANOVA. All p-values < 0.05 were considered to be statistically significant. The mean value of flexural strength of CAD/CAM PMMA was 118.11±5.00 MPa which was almost similar to the flexural strength of PEEK 120±8.0 MPa. Whereas Conventional heat cure PMMA had the least mean flexural strength of 75.4±450 MPa (Table 1, Figure 5).

**Table 1:** One-way ANOVA (analysis of variance) for mean values of impact strength and flexural strength of test samples

	Impact strength (kJ/m^2^)			Flexural Strength (MPa)		
Group	Mean	Standard deviation (SD)	95% confidence interval (CI)	Mean	Standard deviation (SD)	95% confidence interval (CI)
Group I - Control	4.00	0.11	4.222, 3.80	75.4	4.5	70.11, 80.11
Group II - PEEK	10.22	1.25	11.00, 10.543	120	8.0	122.11, 118.45
Group III - CAD-CAM PMMA	9.595	3.313	10.115, 9.500	118.11	5.0	120, 110.50

 

## Discussion

The first resin material was introduced in the year 1937 and many other materials have been introduced since then but no materials are closely related to intraoral soft tissues [1]. PMMA is one of the most commonly used denture base resins but has low physical and mechanical properties due to which it has greater chances of failure when compared to newer materials [1]. The method of fabrication of a complete denture using PMMA also affects its strength [2]. CAD/CAM fabricated dentures yield better physical and mechanical properties than dentures fabricated using injection molding technique followed by the least strength in dentures fabricated using conventional heat cure PMMA mainly because of less error in processing technique [6]. CAD/CAM also enables denture fabrication with lesser weight and lesser resin volume, thus increasing patient comfort. Because of the drawbacks of PMMA, PEEK was introduced in dentistry in the year 1999 [7-9]. Kurtz et al. stated that PEEK has a high potential to be used in dentistry [10]. Various other authors in their studies mentioned that PEEK has a similar elastic modulus to that of human bone, i.e., 3.7-4.0 GPa, and also is chemically inert which makes it a good candidate to be used in place of PMMA [11]. Zoidis et al. in 2015 suggested that PEEK could be of use as an alternative to framework material for removable dentures as they are non-allergic, have low plaque affinity, and are biocompatible [12]. However, there are very few studies present in the literature that have compared PEEK and CAD/CAM PMMA and checked their impact and flexural strength. Shrivastava et al. conducted research to assess a few mechanical characteristics of PMMA and PEEK and concluded that PEEK could be utilized as a material for denture bases with better mechanical properties over PMMA, but it has certain drawbacks [13]. In their article, they have used conventional technique for fabrication which includes many procedural errors. To avoid such errors in our study we have included CAD/CAM denture base resin.

The mean impact strength value was checked using an Izod impact tester. Many factors influence impact strength and fracture characteristics such as the material used, specimen size and position, fabrication technique, the concentration of stress, etc., of which concentration of stress is the primary contributor to denture’s impact failure in the form of grooves, sharp edges, rough surface, depressions or notches, etc. [14].

The mean flexural strength value was checked in a universal testing machine using a three-point bending test which is the gold standard for assessing flexural properties as per ISO 20795-1 for denture base polymers and the international standards for polymer materials [15]. As per the standard, 65MPa is the desired minimum value of flexural strength of denture acrylics and all groups in this study meet the acceptable flexural property requirement for clinical usage [15].

The mean impact strength values of CAD/CAM PMMA and PEEK were 9.5±3.313 kJ/m^2^ and 10.22±1.25 kJ/m^2^, respectively, and the lowest was that of conventional heat cure PMMA, i.e., 4.00±.011 kJ/m^2^. The mean value of flexural strength of CAD/CAM PMMA and PEEK were 118.11±5.00 MPa and 120±8.0 MPa, respectively, and the least was that of Conventional heat cure PMMA, i.e., 75.4±450 MPa.

These observations are in agreement with Al-Dwairi et al., Dubey et al., and Aguirre et al., who also concluded that values of impact and flexural strength of the CAD/CAM resins were higher as compared to those made using the conventional approach [16-18]. Shrivastava et al. similarly concluded from their study that values of hardness and flexural strength of PEEK are more than that of PMMA as a result prostheses with PEEK substructure had a better prognosis and enhanced patient comfort and acceptance [13].

The higher impact strength and flexural values of the CAD/CAM PMMA and PEEK samples as compared to the conventional heat cure samples in this study could be attributed to their higher polymerization degree, which imparts strength to them [19]. The CAD/CAM resin blocks are pre-polymerized to a very large extent, forming a highly condensed resin mass with the least possible porosities [20]. Similarly, the reason for lower values for conventional heat cure PMMA could be due to high temperature during the curing cycle which causes free monomer to boil leading to porosities and surface defects in the denture base resin. These porosities lead to internal stresses and propagation of cracks within the resin ultimately causing decreased strength and density.

Lower physical and mechanical properties of conventional heat cure PMMA led to the development of CAD/CAM denture base resins with better properties. However, they also have certain limitations like higher cost, material wastage, associated learning curve, lack of secondary processing ability, and thermoformability. One of the limitations of this study could be the small sample size which increases the margin of error in the statistical analysis and results.

## Conclusions

CAD/CAM has led to an increase in the use of PEEK in dentistry as its superior mechanical properties and biocompatibility makes it an ideal material for removable prosthesis. From this study, it can be summed up that the CAD/CAM denture base resins whether PMMA or PEEK, have better or superior mechanical properties as compared to conventional PMMA and that PEEK is an excellent material option for the fabrication of complete dentures in term of flexural and impact strength.
